# The Impact of Leadership Programme on Self-Esteem and Self-Efficacy in School: A Randomized Controlled Trial

**DOI:** 10.1371/journal.pone.0052023

**Published:** 2012-12-18

**Authors:** Martin C. S. Wong, Tony C. M. Lau, Albert Lee

**Affiliations:** School of Public Health and Primary Care, Faculty of Medicine, School of Public Health and Primary Care, Chinese University of Hong Kong, Prince of Wales Hospital, Shatin, the New Territories, Hong Kong Special Administrative Region, China; University of Ottawa, Canada

## Abstract

**Background:**

Leadership training programs by experiential learning among adolescents are very popular worldwide and in particular developed countries, but there exists few studies which formally assessed their impact on the psychological well-being of program participants. This study evaluated the effectiveness of leadership training programs on self-esteem and self-efficacy among adolescents.

**Methodology/Principal Findings:**

a total of 180 students of the same grade of one secondary school were randomized into an intervention (n = 50) and a control group (n = 130). The students in the intervention group participated in a 6-month program of leadership training and service learning, while the control group did not participate in any training. Their self-esteem and self-efficacy were assessed by Rosenberg Self-Esteem questionnaire and Chinese Adaptation of the General Self-Efficacy Scale, respectively, before and after the program. Both scales have been recognized internationally as valid and reliable survey instruments to measure these psychological attributes. The scores were compared by Student’s tests according to gender. A total of 180 students were enrolled during the study period October, 2009 to May, 2010. Their mean age was 15.18 years (0.62) and 56.7% were male. Students allocated to the intervention and control group had statistically similar demographic characteristics except gender (male 36.0% vs. 64.6%, p = 0.001). Overall, the self-esteem scores increased by 1.28 and decreased by 0.30 (p = 0.161) while the self-efficacy scores increased by 0.26 and decreased by 0.76 (p = 0.429) in the intervention and control group, respectively. Among female students, the intervention group showed significant improvements in both self-esteem (2.38 vs. −0.24, p<0.001) and self-efficacy (1.32 vs. –0.04, p = 0.043).

**Conclusions/Significance:**

Leadership training program were not found to be effective to enhance self-esteem and self-efficacy in adolescents, except girls who showed modest increase in these outcomes. Future research should assess the reasons why these programs are effective among female.

## Introduction

Self-esteem is a person’s positive or negative attitude toward himself or herself [1], and is closely associated with personality functioning. Among children and adolescents, it is a central concept related to academic achievement, social functioning and psychopathology. Studies indicated that children with low self-esteem were less successful at schools [2], less accepted by their peers [3], and was linked with childhood psychopathology, including anxiety [4], [5], depression [2], [6] and eating disorders [7], [8]. Self-esteem is a primary force that prevents maladaptive adolescent behavior like drug abuse and engagement in damaging peer relationships [9].

Self-efficacy, on the other hand, refers to an individual’s belief in his capacity to execute behaviors necessary to achieve specific performance attainments [10]–[12]. It reflects confidence in the ability to exert control over one’s own motivation, behavior, and social environment. Its perceptions influence the choice of activity, task perseverance, level of effort expended, and ultimately the degree of success achieved [10]. Low levels of self-efficacy in early adolescence adversely affect the potential for attaining academic success, was associated with physical and verbal aggression, and greater emotional irascibility [11].

However, the development of self-esteem and self-efficacy in students should be matched with a supportive learning environment, which should involve various stakeholders of the school [13], [14]. In order to provide opportunities for students to enhance psychosocial development, leadership training programme is one of the typical methods to train students’ confidence and decision-making in many Western countries including Europe [15].

The major goals of leadership training and related school based activities are to guide students to have successful experience in volunteer services and school based moral education programs. Students’ self-esteem could be built up by gaining respect from teachers, parents, classmates, friends, service target groups and the society. Trust and confidence are the key elements in building up students’ self-efficacy [10]–[12]. Positive self-image can also be established if they are well accepted by their teachers and parents [1]–[3].

The philosophy of these programs is based on a strategy – “Service Learning Approach”, which “…is a form of experiential education where learning occurs through a cycle of action and reflection as students work with others through a process of applying what they are learning to community problems and, at the same time, reflecting upon their experience as they seek to achieve real objectives for the community and deeper understanding and skills for themselves” [16]. Several research studies have found evidence that service learning has a positive impact on students in a number of ways, including personal development, efficacy, and identity [17]. Service learning is “a teaching and learning approach that integrates community service with academic study to enrich learning, teach civic responsibility, and strengthen communities” [18].

Recent school-based interventions on students’ resilience levels have reported positive results, including enhancement of self-esteem [19], [20]. In addition, one meta-analysis has evaluated the impact of 213 school-based interventions on enhancing students’ social and emotional learning (SEL). It was found that participants in these SEL promoting programmes had significantly better social and emotional skills, attitude, behavior, and academic performance [21]. The results were further echoed by an international analysis showing these positive changes [22]. Nevertheless, these programmes have interventions which mostly take the formats of teacher- or non-school personnel-administered classes, with or without a parent component and school-wide initiatives. There were few studies which provided solid scientific evidence for the impact of school-based training programmes which involves extensive community service participation and leadership training in strengthening the psychological dimensions of adolescents [23]–[24].

In this context, this study formally evaluated the effectiveness of a school-based leadership training programme on enhancing the levels of self-esteem and self-efficacy among adolescents. We tested the hypothesis that a newly-designed, highly participatory training programme was effective to increase self-esteem and self-efficacy in secondary school students.

## Materials and Methods

This study was approved by the Survey and Behavioral Research Ethics Committee of the Chinese University of Hong Kong. Both the students and their formal guardians participated in this project were informed, with their verbal consent obtained. The surveys were anonymous and only aggregate data were presented. Individual data or personal particulars were kept confidential by researchers. The school principal endorsed this project regarding its operation and planning, agreed the participation of students and parents in this project, as well as data collection using surveys. The ethics committee has waived the requirement of using consent forms as school consent had been sought and none of the survey items contained any sensitive issues. The principal researcher has also kept a file which documented the list of students who have given verbal consent. **During the consent process, all students were thoroughly offered a full description of the program including its duration, interventional components and commitments required. The students were informed that participation in the programme is completely voluntary and refusal would not lead to any penalties.**

### Participants and Study Design

One mainstream secondary school in an underprivileged district of Hong Kong was purposively invited to participate in this study. The district has a median monthly household income of US$2,055, compared with the Hong Kong wide figure of US$2,240 [25]. We included all students in secondary grade 4 of this school (N = 180) during the study period October, 2009 to May, 2010. We adopted the design of a randomized controlled trial. We randomly allocated all students into an intervention group (n = 50) and a control group (n = 130) from computer-generated random numbers with a student regarded as one unit of randomization. The allocation ratio was not 1∶1 since the maximum quota for each programme could accommodate 50 students only. Students in the intervention group received a leadership training programme, including organizing volunteer services and school-based moral and civic activities. All students were enrolled by the class teachers. There were no changes to the methods and trial outcomes after trial commencement. Not having previous data on the estimated changes in the scores, we considered an increase of 2.5 points in self-esteem or self-efficacy being significant. Assume a standard deviation of 4.0 in both the control and intervention groups, and we set the test as two-tailed, alpha = 0.05 and beta = 0.20, the minimum sample size was 40 in each group. We determined to recruit 50 subjects in the intervention group to account for a possible attrition rate of up to 20%. We did not implement any programme for students in the control group. All students who were mentally competent to join the programme were included, as independently assessed by their respective class teachers who were familiar with the mental competence of their students. We regarded that this programme would confer benefits to adolescents at their developing stage, irrespective of their baseline self-esteem and self-efficacy levels.

### Intervention

The leadership training programme aims to improve the capability of the students to learn more effectively, develop creativity, willpower, emotional intelligence, social communication skills and critical thinking. The project was named “Hand in Hand Serves the Community”, targeting mainly to adolescents at the age range of 15–16 years. A series of leadership training activities, volunteer services and school-based moral educational programs were organized to promote students’ self-image and confidence. This practice-based programme was designed by educational expert with extensive experience in adolescent training, while qualified trainers on leadership skills were invited as the programme coaches and coordinators. Its activity components have been designed, further discussed and finalized among teaching professionals to suit the target participants in the age range of 15–16 years, taking into account local situations. The theme of this project is ***“Stride over yourself & Contribute to the society”*** **.** It could provide opportunities for students to experience success, failure, reshape themselves, rebuild their self-confidence and trust with peers, parents and teachers. The rationale of this project was to raise students’ confidence and build up positive self-image by carrying out leadership training and performing volunteer services, with an ultimate aim to build up their self-esteem and self-efficacy. Students could take this opportunity to show their concern and provide services for the disabled children (e.g. those with neuro-developmental disorder, neurological and degenerative diseases, scoliosis, spinal deformities and cerebral palsy) and single living seniors residing in underprivileged regions. The total contact hours were approximately 20 hours, with 4 hour of activity participation monthly for a 5-month training period. The implementation was the same for all students in the intervention group, and all 50 participant students attended the same class. The activities in the programme were designed to enhance students’ organizational capability, problem solving skills, team building techniques, fostering of care and concerns towards others, sharing of successful experiences, and build up expertise on activity design among the programme participants. The details of this training programme were described in **Table 1**.

**Table 1:** Details of the leadership training programme.

Phase	Activity	Period	Objectives
**1. Recruitment**	Student recruitment	October, 2009	A total of 50 secondary grade 4 students were randomly recruited as volunteers to form Volunteer Social Service Team
**2 Volunteer training**	Group Meetings and Training	October, 2009–March, 2010	1. One regular training session was organized monthly for 5 months to provide challenging tasks for students to complete.
			2. To train students on techniques regarding how to plan and organize a volunteer service especially for children with neuro-developmental disorder, neurological and degenerative diseases, scoliosis, spinal deformities and cerebral palsy.
	Volunteers’ Training Camp	October, 2009	3. To improve students’ problem solving and team-building skills.
			4. To develop students’ patience in looking after children and single living seniors.
			5. To build up students’ self-esteem, self-efficacy and resilience by sharing of successful experience in adversities.
**3. Experience and action period**	Friendship Building withchildren and single living seniorsin an underprivileged region	October, 2009	1. Students could know more about the learning pattern of the severe mentally retarded children and the special facilities/equipments used for physical and psychological treatment.
			2. Students could learn how to design special activities for the severe mentally retarded children and exposed to the difficulties they may face when organizing services.
	Voluntary Service PracticeCamp	November, 2009	3. Students could invite their classmates to join this program and serve the community together.
	Festival Greeting – HappyChristmas Party in Hong KongRed Cross Princess AlexandraSchool	December, 2009	4. Students could learn how to express their concern, love and support to the children and their families in the most empathetic manner.
	Visit to the Hong Kong RedCross Princess Alexandra School	March, 2010	5. To develop students the sense of caring to other people in need, regardless of their backgrounds
	Po Leung Kuk Elder Academy	October, 2009–May, 2010	6. Students can transmit the message of love to disadvantaged groups and advocate peace and friendship in the society.
4. **Evaluation and Experience Sharing**	Graduation ceremony of “Stride over yourself & Contribute tothe society” Service	May, 2010	1. To consolidate the life experiences of students in this program.
			2. To provide opportunities for students to express their feelings on communication with the children, single living seniors and children with neuro-developmental disorders
			3. Students can evaluate their performance in this program. (E.g. self-esteem, self-confidence, self-belonging and self-image)
			4. To reinforce students’ self- confidence in the difficulties and challenges they have to face in their lives.
			5. To promote the sense of commitment and care about others’ needs.

### Primary Outcomes

Self-administered surveys were used to assess self-esteem and self-efficacy before and after the programme. The Chinese version of the Rosenberg self-esteem questionnaire was used to assess self-esteem [26]. It is a self-report measure of global self-esteem, and the scale was validated with 10-questions having a score range from 0 to 30. A higher score indicates a higher level of self-esteem. The items are designed on a four-point Likert scale ranging from “strongly agree” to “strongly disagree”. It has been validated for use in both male and female adolescents, with a Cronbach’s alpha of approximately 0.80. This Rosenberg self-esteem scale has been used in previous studies among college students and adolescents [27], and is considered a reliable and valid quantitative tool for assessment of self-esteem [28].

The Chinese Adaptation of the General Self Efficacy Scale questionnaire [29] was used to assess self-efficacy. The scale is designed for the general adult population, including adolescents. It has 10 questions with a score range of 10–40; the higher the scores, the higher is the efficacy of the student. The construct of Perceived Self-Efficacy reflects an optimistic self-belief [30]. Its Cronbach’s alpha ranged from 0.76 to 0.90, and the scale has been extensively used in different population groups including adolescents in schools [31].

### Statistical Analysis

We used the Statistical Package for Social Sciences (SPSS) version 15.0, Chicago, Illinois) for data entry and analyses. The dependent variables were self-esteem and elf-efficacy. The Shapiro-wilk test was used to test for normality distribution of the outcome difference. The baseline characteristics collected included the students’ age, gender, median monthly Household income, housing type (public vs. private), the presence of long-term medical diseases, the use of long-term medications, and the number of sick leave days in the past one year. The monthly household income reported here (<HK$5,000 [US$642]; HK$5,000–9,999 [US$642–1,284]; ≥ HK$10,000 [US$1,284] are commonly used income ranges in school surveys locally in Hong Kong. Students in the control and intervention groups were compared with respect to their baseline demographic characteristics, using chi-square tests of independence and student’s t-tests for categorical and continuous variables, respectively. A gender-stratified analysis was performed to explore whether there exists gender differences in the changes of the self-esteem and self-efficacy scores.

We then compared the changes in the scores between the control and intervention groups using student’s t-tests among all participants; male; and female students. All p values ≤0.05 were regarded as statistically significant.

## Results

When the demographic characteristics of the control and intervention groups were compared, there were no statistically significant differences between the two groups with respect to age, median monthly household income, type of housing, long-term medical diseases, use of medications, the number of days reporting sick leaves in the past year, as well as the self-esteem and self-efficacy scores except gender (male 64.6% vs. 34.0%, respectively, p<0.001). There were no missing data in this study.

From Shapiro-wilk test, the differences in scores before and after the programme of the intervention and control groups for self-esteem (p = 0.304 and 0.348, respectively) and self-efficacy (p = 0.189 and 0.330, respectively) were normally distributed. Overall, the scores of self-esteem and self-efficacy increased by 0.13 and decreased by 0.48, respectively (**Table 2**). The intervention group was found to have improvements of both self-esteem (1.28) and self-efficacy (0.26) scores while the control group showed a decrease in both scores. However, the differences in these changes were not found to be statistically significant (p = 0.161 and 0.429, respectively). Gender-stratified analyses showed that female students had significantly increased scores in both dimensions of self-esteem (+2.38 vs. −0.24, p<0.001) and self-efficacy (+1.32 vs. −0.04, p = 0.043) (Table 3). There were no significant differences in both scores between the control and intervention groups among male students (self-esteem −0.67 vs. −0.33, p = 0.610; self-efficacy −1.62 vs. −1.05, p = 0.481), respectively. However, both scores decreased among males.

**Table 2:** Participant characteristics.

	Control Group(N = 130)	Intervention Group(N = 50)	p-value
**Age (mean+S.D.)**	15.18 (0.64)	15.20 (0.57)	0.823
**Gender**			
Male	84 (64.6%)	18 (36.0%)	0.001
Female	46 (35.4%)	32 (64.0%)	
**Median monthly Household Income**			
≤ US$642/month	5 (3.8%)	4 (8.0%)	0.221
US$642–1,284/month	26 (20.0%)	14 (28.0%)	
≥ US$1,284/month	99 (76.2%)	32 (64.0%)	
**Housing type**			
Public	82 (63.1%)	30 (60.0%)	0.514
Rent	13 (10.0%)	3 (6.0%)	
Self-owned	35 (26.9%)	17 (34.0%)	
**Long term medical disease**			
Yes	4 (3.1%)	4 (8.0%)	0.298
No	126 (96.9%)	46 (92%)	
**Long term drug use**			
Yes	2 (1.5%)	2 (4.0%)	0.316
No	128 (98.5%)	48 (96%)	
**No. of days of sick leaves in the past year**			
0–1 day	107 (82.3%)	39 (78.0%)	0.508
≥2 days	23 (17.7%)	11 (22.0%)	
**Baseline scores**			
Self-esteem	17.68 (4.55)	17.78 (4.68)	0.896
Self-efficacy	25.48 (4.82)	25.48 (5.82)	1.000

All percentages were across columns.

**Table 3:** Changes in the students’ scores of self-esteem and self-efficacy before and after the intervention.

	All participants (N = 180)			Control Group (n = 130)			Intervention Group (n = 50)			Effect Size	p-value
	Pre	Post	change	Pre	Post	change	Pre	Post	change		
**Self Esteem**											
Overall	17.71 (4.57)	17.84 (4.22)	**+0.13**	17.68 (4.55)	17.38 (4.40)	−**0.30**	17.78 (4.68)	19.06 (3.46)	**+1.28**	**+1.58**	**0.161**
Male	18.08 (5.02)	17.69 (4.06)	−**0.39**	17.77 (5.00)	17.44 (4.09)	−**0.33**	19.50 (5.00)	18.83 (3.79)	−**0.67**	−**0.34**	**0.610**
Female	17.22 (3.89)	18.05 (4.43)	**+0.83**	17.50 (3.62)	17.26 (4.95)	−**0.24**	16.81 (4.27)	19.19 (3.32)	**+2.38**	**+2.62**	**<0.001**
**Self Efficacy**											
Overall	25.48 (5.10)	25.00 (5.51)	−**0.48**	25.48 (4.82)	24.72 (5.95)	−**0.76**	25.48 (5.82)	25.74 (4.12)	**+0.26**	**+1.02**	**0.429**
Male	26.33 (5.28)	25.10 (6.00)	−**1.23**	26.07 (4.97)	24.92 (6.31)	−**1.05**	27.56 (6.56)	25.94 (4.32)	−**1.62**	−**0.57**	**0.481**
Female	24.36 (4.65)	24.87 (4.83)	**+0.51**	24.39 (4.38)	24.35 (5.27)	−**0.04**	24.31 (5.10)	25.63 (4.07)	**+1.32**	**+1.36**	**0.043**

The numbers refer to the means (standard deviations in parentheses).

## Discussion

From 180 secondary students, we found that the overall self-esteem and self-efficacy scores increased after implementation of the programme. The differences in the changes of these scores were statistically significant only among female students. We concluded that this school-based leadership training programme was effective to enhance self-esteem and self-efficacy among female adolescent students. Nevertheless, the absolute differences in the score changes were modest (2.62 points in self-esteem and 1.36 points in efficacy).

Studies in a wide range of western countries have determined that adolescent females, on average, have a lower sense of self-esteem than adolescent males [32], [33]. Boy’s self-esteem can be affected by contradictory societal messages – on the one hand to appear to be strong and on the other to be emotionally expressive [34]. In the present study, female but not male students were found to benefit from the training programmes. A recent longitudinal study among white adolescents in the Midwest applied a social structure and personality perspective to examine variations in self-esteem and mastery trajectory by gender [35]. It was concluded that self-esteem increases at a faster rate among girls than boys during high school. Rebelliousness, which exits gender disparity, has also been demonstrated to be an independent factor associated with low self-esteem [36]. **Nevertheless, there exists many cultural and geopolitical factors which might not allow direct comparison between studies conducted in Western countries and the present one.** Future studies should explore why female students showed significant changes in both scores whilst male students had decreased scores in both self-esteem and self-efficacy. It is unknown whether these training programmes were not emotionally conceivable by male students due to the programme design, or a chance finding with a short period of follow-up.

The explanation why experiential learning could lead to better self-esteem and self-efficacy was well supported by existing literature. The natural human desire to help others is based on the belief that “when a person gives and becomes valuable to others, feelings of self-worth are increased and a more positive self-concept is built” [37]. Youths get the powerful feeling of being engaged in “something beyond themselves” [38], which can have a transformative effect on behaviors and feelings. When youths' contributions to helping others are acknowledged, youths feel proud and feel as if their life has a purpose [23].

Research suggests that community participation and service learning can improve educational attitudes and performance, decrease risk-taking behavior, help youths develop positive relationships [24], and improve youths’ social capital and social networks [39]. Although community service focuses on fulfilling a community need, “service learning” focuses on fulfilling a community need and enlivening and expanding the material taught in school [40]. Service learning has been acclaimed as a way to add context to coursework and build concrete skills [40], increase students’ motivation because they know their efforts will benefit others [41], and help students think critically about meaningful real-life experiences [41].

Service Learning connects to learning outcomes. Students have the opportunity to apply their experiences to real-life situations through the reflective process. By providing students opportunities to learn by preparing, leading, and reflecting upon their service experiences, they ultimately create a reciprocal learning experience between them and the community.

This is a randomized controlled trial and we found that the majority of the participant characteristics were similar between the control and intervention group before subject enrollment. There were no subject drop-outs or any attrition in any sessions of this training programme. These programmes include team-building, service learning and project planning initiatives, and it is anticipated that only the participants in the intervention group could receive direct benefits. However, some of its limitations should be addressed. First, this study captured only short-term outcomes of self-esteem and self-efficacy within a time frame of 6 months, and the sustainability of the positive changes due to the intervention was yet to be evaluated. Although the measures of self-esteem and self-efficacy have relatively satisfactory psychometric properties (reliability ranged from 0.76–0.90), **the self-reported nature of the surveys might introduce some forms of ascertainment bias, and there have been few studies evaluating whether the scales used in the present study were sensitive to change over time.** Also, there is a remarkable gender imbalance between the control and intervention group, although there exists no statistical differences in all other demographic variables between the two groups. The randomization allocation used in this study is 130∶50 due to the maximum quota of 50 students for programme enrollment and this is not as desirable as a 1∶1 allocation ratio. In addition, these two outcome measures represent only part of the many psychosocial parameters in adolescent development, and we have not measured the other components which could be influenced by the training programme. These include self-concept, school bonding, beliefs about mutual help, positive social behavior and conduct problems [21]. Furthermore, one might argue that interaction between the intervention and control groups could take place; there is no strategy implemented in this programme to prevent communications between students of different study groups about their experiences throughout the study period. So, controls may very well have been exposed indirectly to the intervention. However, contamination as a potential problem could be minimized by the highly participatory nature and great degree of self-involvement among programme participants, who cannot influence the control group much by directly sharing the programme content to the non-participants. **Finally, there are some potential confounding factors where we have not controlled for, including ethnicity, culture, societal role, experiences of abuse, academic success, self-motivated community participation which might influence the outcome measures, although randomization could theoretically balance these factors between the two groups.**

In summary, this study reported no positive changes in self-esteem and self-efficacy among secondary school students, but we found modest positive improvements among female students on secondary analysis. Previous evaluations have found that one-third to one-half of adolescents struggle with low self-esteem, especially in early adolescence [42], [43]. Future studies should assess long-term impacts of these programmes in different age groups, measure more psychosocial dimensions as study outcomes, and evaluate the reasons why the effectiveness of these programme influenced female students to a greater extent.
